# Optimizing GPT-5 for Operation-Procedure-Code-Extraction from Operative Reports in Meningioma Surgery: Feasibility and Comparison of Context-Enhancements

**DOI:** 10.1055/a-2913-8450

**Published:** 2026-07-23

**Authors:** Sebastian Lehmann, Florian Wilhelmy, Frederic V. Schwaebe, Nikolaus von Dercks, Erdem Güresir, Johannes S. Wach

**Affiliations:** 1 Neurosurgery 39066 University Hospital LeipzigLeipzigSachsenGermany; 2 Medical Management 39066 Universitätsklinikum LeipzigLeipzigSachsenGermany

**Keywords:** OPS-coding, clinical information systems, process management tools, clinical decision support, artificial intelligence, GPT-5, meningioma

## Abstract

**Objective:**

In our recent study we showed that GPT's ability to perform OPS-code extraction from operational reports was equivalent to coding neurosurgeons. In this study we aim to evaluate the effect of context enhancement on GPT-5's code extraction abilities.

**Methods:**

We provided OpenAIs GPT-5 with 100 operational reports (OR) of patients that underwent meningioma surgery. A chat prompt was generated instructing GPT to generate the correct OPS coding. Five groups were formed and provided with different context-enhancing modalities: 1. No additional context (GPT-5s), 2. the current OPS-catalogue (GPT-5o), 3. specified rules on Meningioma coding (GPT-5r), 4. code-triggering sample phrases based on 50 additional ORs (GPT-5e) and 5. all enhancements combined (GPT-5c). We analyzed code-extraction abilities, mistakes and hallucinations.

**Results:**

Non context enhanced GPT-5s showed the lowest rate of correct coding (44%) and highest number of hallucinations (105) and total mistakes (132). GPT-5o showed identical accuracy to GPT-5s (44%, *p* = 1.0), but fewer hallucinations (51, *p* = 0.002) and mistakes (78, *p* = 0.008). All other models were significantly superior to GPT-5s and GPT-5o in accuracy (GPT-5r 79%, GPT-5e 70%, GPT-5c 86%, *p* < 0.001), hallucinations (GPT-5r 8, GPT-5e 2, GPT-5c 6, *p* < 0.001) and mistakes (GPT-5r 21, GPT-5e 35, GPT-5c 14, *p* < 0.001). Highest coding accuracy was achieved by GPT-5c (GPT-5c vs GPT-5r, *p* = 0.143, GPT-5c vs GPT5e *p* = 0.008). GPT-5r and GPT-5e performed equally regarding accuracy, GPT-5r produced fewer mistakes ( *p* = 0.034), while GPT-5e hallucinated less ( *p* = 0.057).

**Conclusion:**

We show, that specific context enhancement significantly improves GPT-5s ability to perform OPS-code extraction from operational reports, while significantly lowering mistake- and hallucination rates.

## Background and Significance

The medical billing system in Germany is based on categorizing patients into diagnosis-related groups (DRGs). 1 In surgical disciplines, operation procedure classification-coding (OPS system) is the main influencing factor for DRG-grouping. 2 Coding is performed based on the OPS-catalogue, published and annually updated by the Federal Institute for Drugs and Medical Devices. 3 OPS-coding allows for a precise representation of the procedure performed, accounting for the associated effort and material costs. 4

In clinical practice, coding is performed by the operating surgeon and optimized by professional coding assistants employed by the hospital. 4 After case closure and before payout of the calculated revenue, coding is reviewed by the medical service of health insurance providers (MDK). 5 Precise and correct OPS-coding, therefore, forms the foundation of an economically successful surgical department.

In the era of increasing use of artificial intelligence (AI), an increasing number of tasks are transferred to AI agents. 6 7 For medical use, AI has already shown effects across a broad field ranging from research, diagnostics, and training to optimizing workflows and patient safety. 8 Under continuous economic pressure, outsourcing tasks to AI offers a new opportunity for process optimization and cost reduction. 9 10 In medical coding, research is currently mainly focused on ICD and DRG coding, already showing promising results. 11 12 Various approaches can be utilized to extract information from text-based primary sources. Rule-based systems rely on predefined decision rules and are characterized by hardware efficiency. 13 However, the fixed decision path offers limited flexibility when confronted with varying linguistic expressions. Hybrid NLP pipelines combine rule-based systems with machine-learning mechanisms, improving generalizability while also increasing integration and maintenance effort. 14 LLMs are well suited to capture semantic relationships across highly variable formulations and can consistently process relevant information over longer text passages. 15

This study focuses on the publicly available and accessible large language model (LLM) GPT (version GPT-5), developed by OpenAI. 16

With improved reasoning abilities, GPT-5 has already been shown to exceed human experts in understanding and reasoning regarding multimodal medical reasoning benchmarks. 17 We also know that the analytic abilities of LLMs are influenced by the level of context provided. 18

To the best of our knowledge, the application of LLMs to OPS-coding has not yet been specifically investigated in the published literature.

In our previous study, we compared untrained context-enhanced GPTs' OPS-coding abilities with those of human coders, in which GPT performed on par with coding neurosurgeons. 19 Here, a coding accuracy of 71 to 83% was achieved by the LLM. However, a relevant rate of content-related mistakes (10–14%) and data hallucinations (7–15%) was observed.

## Objectives

Building on our previous feasibility work, the primary objective of the present study was to compare different transferable context-enhancement modalities regarding their effect on case-level OPS-code extraction accuracy from operative reports (OR). We hypothesized that structured, domain-specific context enhancement (CE) would improve coding accuracy and reduce hallucinations compared with no CE or catalogue-only support.

## Methods

We collected the ORs of 150 patients who underwent meningioma surgery at our institute between 2023 and 2024. A total of 100 ORs were assigned for LLM analysis, 50 ORs for CE. Sample size was determined based on feasibility and on the effect sizes observed in our previous study, reproducing the underlying setting. Based on our previous analysis of discordant proportions in LLM performance in paired comparison, a post hoc sample size calculation for McNemar's test was performed. Assuming a two-sided α level of 0.05 and 80% power, the sample size required was estimated at approximately 97 paired observations.

There was no overlap between these datasets, ensuring a strict separation between CE and validation. The cutoff for assignment was made after 50 ORs in chronological order regarding the date of surgery. Throughout the study period from 2023 to 2024, the OPS-catalogue remained unchanged regarding coding relevant to this analysis. The ORs were anonymized. Data regarding neuromonitoring that was only accessible via the patient file was added to the OR. The finalized in-hospital coding was used as a benchmark defining correct coding. Each OR was reviewed, and the correct OPS-coding was defined. In cases of divergent, yet formally correct coding, a consensus-based individual decision was made in consultation with our hospital coders.

OpenAI's current LLM, GPT-5, was provided with the chat prompt and one of 100 ORs. All inferences were conducted from August 1, 2025, to September 30, 2025, via the ChatGPT web interface (OpenAI). For each OR, a standardized prompt ( Supplementary Fig. 1 ) was manually entered into a new chat session to avoid cross-case contamination. CEs were uploaded as separate files depending on group assignment. The model's first complete response was recorded without regeneration or iterative refinement.

**Fig. 1:**
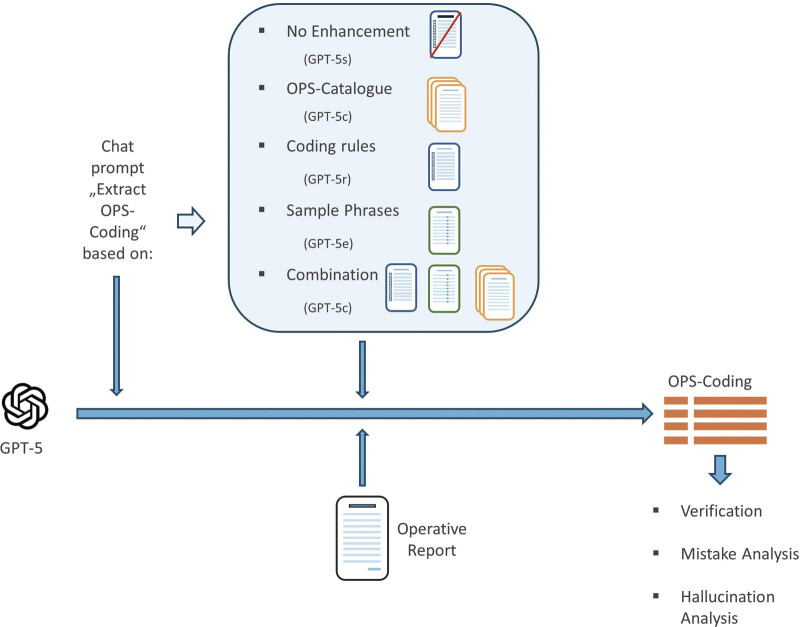
Depiction of the workflow and analyzed groups.

For support and CE, different files were attached to base the analysis on. According to the attached files, we formed five groups for analysis ( Fig. 1 ):

▪ GPT-5 solo (GPT-5s): GPT-5 without CE (GPT-5s).
▪ GPT-5 OPS (GPT-5o): the 2025 OPS catalogue containing codable OPS-codes and official comments.
▪ GPT-5 rules (GPT-5r): a set of rules further defining inclusion and exclusion criteria for the most prevalent OPS-codes; see Supplementary Material ( Text S1 , available in the online version only).
▪ GPT-5 example (GPT-5e): a list of phrases that triggered coding in previous OR (50 OR extracted for CE), in addition to the respective applicable OPS-codes; see Supplementary Material ( Text S2 , available in the online version only).
▪ GPT-5 combined (GPT-5c): all files combined.

The Primary target was correct OPS-coding. Correct coding was achieved when all codes representing the approach and procedure were correctly represented. Additionally, coding was only considered correct if applicable supplementary codes for microsurgery, neuromonitoring, neuronavigation, duroplasty, and others were correctly coded. If multiple possible competing codes were applicable, coding was considered correct, as long as the section (e.g., neuromonitoring) was represented.

As a secondary target, we investigated the LLM's mistakes. We recorded content-related mistakes as well as hallucinations. Mistakes were divided into vital mistakes involving the main procedure or approach coding that could subsequently lead to MDK-rejection and nonvital mistakes that may lead to a revenue decrease by incomplete depiction in the performed procedure. Cases of chaotic miscoding, including exclusively erroneous coding with no plausible relation to the content of the OR, were defined as “LLM-failure.” The number of correctly coded ORs was recorded, as well as the total number of mistakes and the type of mistake. For hallucinations, we defined misuse and nonsense hallucinations. Misuse hallucinations were defined as incorrect matching of existing OPS-codes and content. Nonsense hallucinations were defined as the use of nonexisting OPS-codes.

For statistical analysis, data were gathered in an SPSS (IBM Corp., released 2023. IBM SPSS Statistics for Windows, Version 29.0.2.0 Armonk, New York, United States: IBM Corp) database. Dichotomous variables were analyzed using the McNemar test for related samples. Count data per-case variables were analyzed using paired two-sided *t* -testing.

## Results

Of the 100 patients with meningiomas that were attributed to LLM analysis, 36 were located at the convexity, 10 falcine, and 6 in the central region. Twenty-nine meningiomas were located at the sphenoid wing, 17 in the frontobasal region, and one at the petrous bone. Two patients underwent recurrent surgery. All procedures were performed microsurgically, neuromonitoring was used in 54%, and neuronavigation in 87%. Duroplasty was performed in 93% of cases. Cranioplasty with bone cement implant was performed in 2%; in one case, a vessel was clipped to control damage to vascular structures. One patient underwent intraoperative CPR. The baseline characteristics of the ORs used for validation closely matched those of the ORs used for CE generation. However, neuromonitoring was applied significantly more frequently in the validation cohort (see Table 1 ).

**Table 1:** Baseline meningioma characteristics for CE-arm (50 meningioma-OR) and validation-arm (100 meningioma-OR), *p* -Values calculated by Fisher's exact test and two-tailed *t* -test

Meningioma characteristics	CE-arm (%)	Validation-arm (%)	*p* -Value
Total	50	100	
Convexity	15 (30)	36 (36)	0.58
Sphenoid wing	13 (26)	29 (29)	0.85
Frontobasal	8 (16)	17 (17)	0.63
Falcine	7 (14)	10 (10)	0.59
Central region	2 (4)	6 (6)	1.0
Petrous bone	3 (6)	1 (1)	0.11
Sphenoorbital	2 (4)	0	0.11
Recurrent surgery	1 (2)	2 (2)	1.00
Microsurgery	50 (100)	100 (100)	1.00
Neuromonitoring	15 (30)	54 (54)	0.006
Neuronavigation	47 (94)	87 (87)	0.27
Duroplasty	47 (94)	93 (93)	1.00
Cranioplasty	4 (8)	2 (2)	0.10
Clipping	0	1 (1)	1.00
CPR	0	1 (1)	1.00
OR word-count (mean)	477 (SD: 203.9)	498.5 (SD: 192.0)	0.91

### Coding Accuracy

**Fig. 2:**
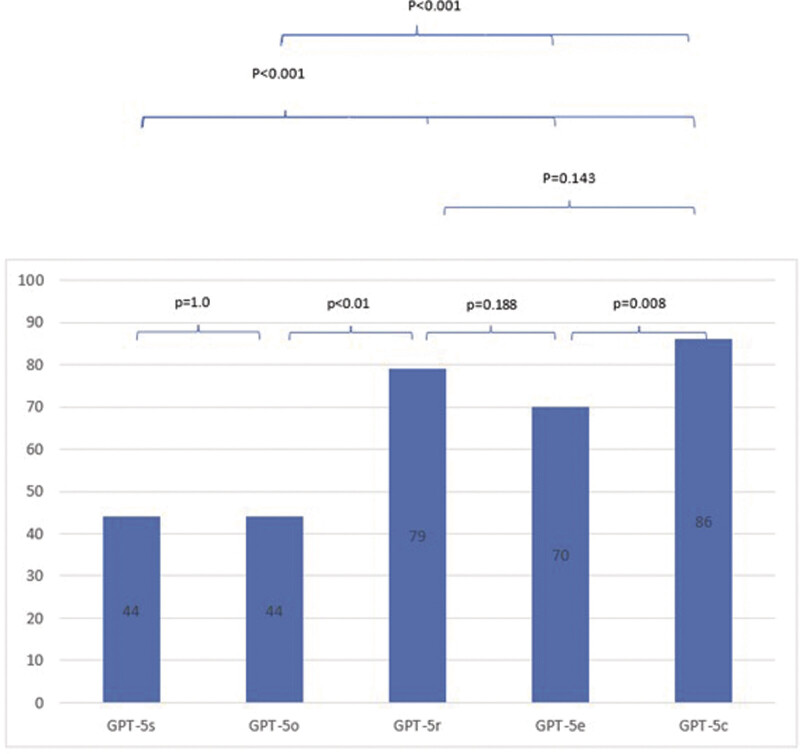
Comparison of correct coding rate for LLM groups, p-measure by McNemar testing.

GPT-5s and GPT-5o showed identical accuracy (44/100 correct codes), followed by GPT-5e (70/100) and GPT-5r (79/100). The highest coding accuracy was achieved by GPT-5c (86/100). Compared with GPT-5s and GPT-5o, statistically significant superiority of correct coding was observed for GPT-5r ( *p* ≤ 0.001), GPT-5e ( *p* ≤ 0.001), and GPT-5c ( *p* ≤ 0.001). No significant difference was observed in the comparison of GPT-5r versus GPT-5e ( *p* = 0.188), as well as GPT-5r versus GPT-5c ( *p* = 0.143). GPT-5c, however, showed significantly superior coding to GPT-5e ( *p* = 0.008); see Fig. 2 .

#### Mistake Analysis

GPT-5s and GPT-5o featured identical rates of overall incorrect coding of OR, regardless of the number or nature of mistakes (66/100). GPT-5s, however, showed a significantly higher mean number of mistakes per OR than GPT-5o (1.32 ± 2.08 vs. 0.78 ± 0.99, *p* = 0.008). GPT-r, GPT-5e, and GPT-5c were observed to be significantly less prone to mistakes (GPT-5r 0.21 ± 0.41, GPT-5e 0.35 ± 0.56, GPT-5c 0.14 ± 0.35) compared with GPT-5s and GPT-5o ( *p* ≤ 0.001). GPT-5r made significantly fewer mistakes than GPT-5e ( *p* = 0.034), while no difference was observed compared to GPT-5c ( *p* = 0.09). GPT-5c performed with significantly fewer mistakes than GPT-5e ( *p* < 0.001); see Table 2 .

**Table 2:** Mistakes by GPT-5-versions

	Wrongly coded OR	Total mistakes	Mean number of mistakes/OR	SD of mistakes/OR
GPT-5s	66/100	132	1.3	2.08
GPT-5o	66/100	78	0.78	0.99
GPT-5r	21/100	21	0.21	0.41
GPT-5e	30/100	35	0.35	0.56
GPT-5c	14/100	14	0.14	0.35

Note: Wrongly coded OR: number of ORs with any case of mistake, regardless of nature or count of the mistake; total mistakes = count of mistakes across all ORs; mean number and standard deviation (SD) of mistakes per OR.

**Fig. 3:**
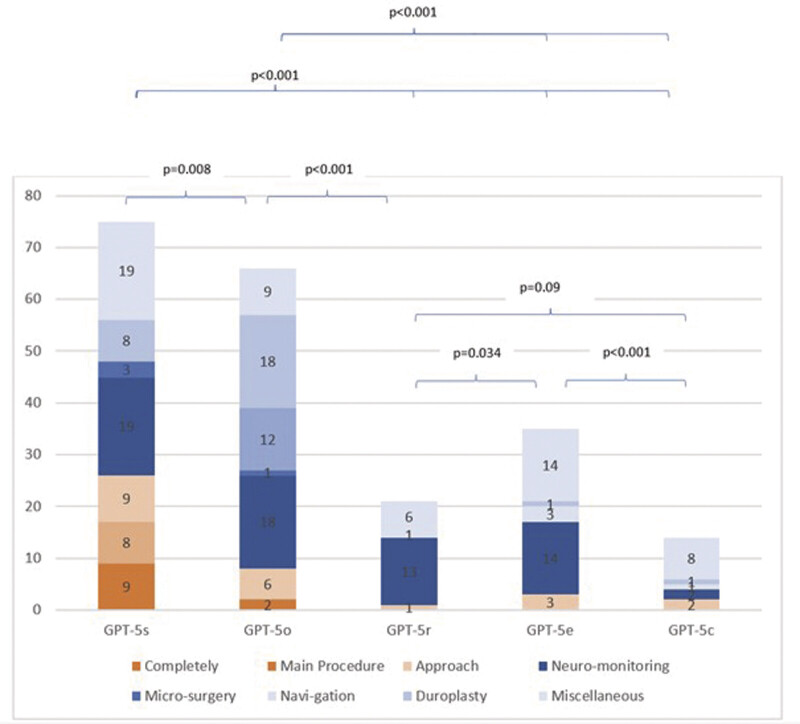
Total mistakes and mistake distribution, orange: vital mistakes, blue: nonvital mistakes. *p* -Values from paired two-sided tests comparing per-case error counts across models.

The distribution of mistakes shows a high rate of LLM failure (9%) that occurs only in GPT-5s. GPT-5s features the most vital mistakes (26%), compared with GPT-5o (8%), GPT-5r (1%), GPT-5e (3%), and GPT-5c (2%). The most common nonvital mistakes were wrongly- or noncoded neuromonitoring (2–19%), wrongly-coded duroplasty (0–18%), and miscellaneous (6–19%), which involved coding for drain placing, bone removal, re-insertion of bone fragment, CPR, and clip-placement that are not typical for meningioma coding; see Fig. 3 .

#### Hallucinations

**Fig. 4:**
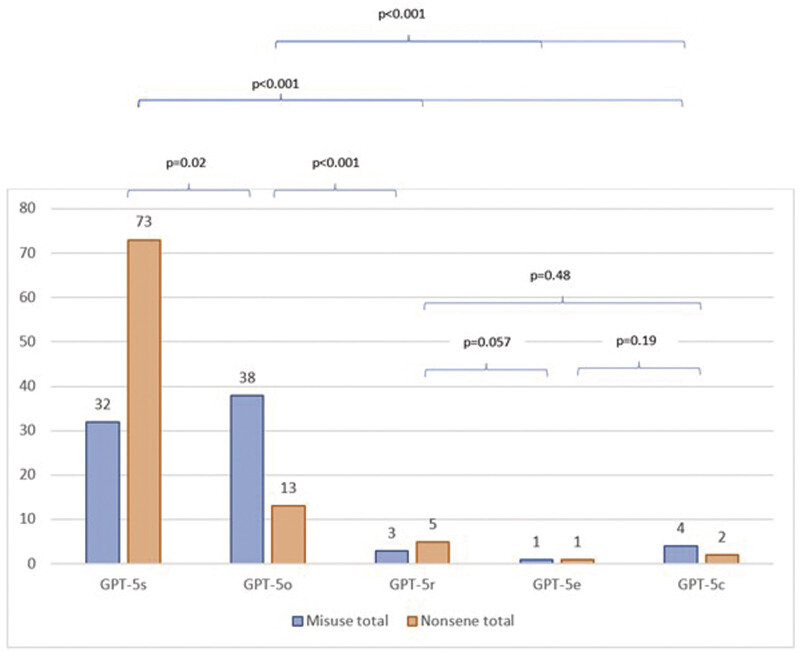
Hallucinations in different LLM groups, divided into misuse (wrong interpretation of existing code) and nonsense (nonexisting code) in total and %. *p* -Values from paired two-sided tests comparing per-case error counts across models.

Among the observed mistakes, a relevant count was attributed to hallucinations. Most total hallucinations were observed in GPT-5s (37%, total 105), followed by GPT-5o (38%, total 51, SD = 2.01, *p* = 0.02). Significantly less hallucinations were observed for GPT-5r (8%, total 8, SD = 2.14, *p* < 0.001), GPT-5e (2%, total 2, SD = 2.11, *p* < 0.001) and GPT-5c (6%, total 6, SD = 2.14, *p* < 0.001), see Fig. 4 . No significant differences were observed in hallucination rate in GPT-5r versus GPT-5e (SD = 0.31, *p* = 0.057), GPT-5r versus GPT-5c (SD = 0.28, *p* = 0.48) and GPT-5e versus GPT-5c (SD = 0.28, *p* = 0.19). While in the occurrence of hallucination per OR, misuse hallucinations appear more frequently (13% vs. 6.2%), a marginal difference is observed in the total numbers of misuse- and nonsense-hallucinations (15.6/100 OR vs. 18.8/100 OR).

## Discussion

In our study, we compared the impact of different modalities of CE on GPT-5′s ability to extract OPS-codes from ORs. No CE (GPT-5s) led to poor results with a high rate of mistakes and hallucinations. While providing the uninterpreted OPS-catalogue (GPT-5o) did not lead to a reduction of correctly coded cases ( *p* = 1.0), fewer total mistakes ( *p* = 0.008) and hallucinations ( *p* = 0.02) were observed. The provision of detailed rules (GPT-5r) or sample phrases (GPT-5e) was observed to significantly raise coding correctness while drastically reducing total mistakes and hallucinations ( *p* < 0.001). Between GPT-5r and GPT-5e, no differences in correct coding were shown ( *p* = 0.188). However, GPT-5e generated fewer hallucinations ( *p* = 0.057) while GPT-5r featured fewer total mistakes ( *p* = 0.034). Best results were achieved in combination with the OPS-catalogue, rules, and sample phrases, GPT-5c.

Most research published on LLM code extraction has mainly focused on diagnostic codes in the ICD-10 system. In a pilot study, a coding assistant was developed by Puts et al showing promising results. 20 In ICD-based clinical event classification, GPT-4 has already been observed to outperform conventional ICD coding systems. 12 Feng et al showed performance comparable to state-of-the-art methods in the DRG-system using GPT-4o. Provided with interventional reports, GPT showed convincing information extraction abilities in patients with ischemic stroke. 21 In practical application, the MOMO-simulation tool using machine-learning algorithms to suggest likely case-individual ICD-coding and coding-combinations is already used in several hospitals. 22 To our knowledge, the application of LLMs to OPS-coding has not yet been investigated in the published literature.

In complex, domain-specific tasks, the performance of LLMs is influenced by both their training and the contextual information provided. 23 Our data show that in nontrained, non-CE LLMs, code extraction abilities are poor. We know that RAG-enhancement (Retrieval-Augmented Generation), where context is provided in different modalities and stages, can improve language processing. 24 The implementation of guidelines was shown to outperform a plain LLM in medical decision-making using a multi-step approach. 18 Domain-specific fine-tuning was shown to greatly increase the LLM performance. 25

In our previous study, GPT was provided with CE in the form of the OPS-catalogue in addition to a list of frequently used codes. This list, however, is hospital-specific, which leads to issues regarding the transferability of the previous results. Our current analysis more clearly depicts insufficient code-extraction abilities in the absence of CE (GPT-5s), as GPT-5s show the highest number of mistakes across all tested models, including CE models from our previous analysis. We also demonstrate the increase of correct code extractions combined with a significant decrease of total mistakes and hallucinations in the application of domain-specific CE designed with a high level of detail (GPT-5r/e/c). A possible explanation for the superiority of coding rules (GPT-5r) over sample phrases (GPT-5e) is the improved transferability to previously unscripted scenarios. However, providing the OPS-catalogue alone does not result in sufficient code-extraction rates. We believe this attributes to the descriptive character of the OPS-catalogue, lacking information for interpretability. In comparison to OPS-coding documented by surgeons (69%) in our previous study, GPT-5r (79%) and GPT-5c (86%) demonstrate superior performance. Compared with our previous study, where we investigated the code extraction abilities of GPT-4 based on the same dataset, 19 we observe a slight improvement of GPT-5. However, comparison is limited due to different CEs and stricter criteria for correct coding in our current study.

Our study confirms the influence of CE on code-extraction abilities, with correct code extractions rising from 44 to 86%. GPT-5o produced fewer mistakes and hallucinations; however, it did not improve the overall rate of correct code extraction. GPT-5r, GPT-5e, and GPT-5c that were provided with detailed and topic-specific CE outperformed GPT-5s and GPT-5o significantly. However, providing a large amount of context data is reported to lead to the “lost in the middle” problem, where the influence of provided data is variable and dependent on its position in the document. 26 A possible solution is weighting and a clear separation of documents. 27 In this study, GPT5c was provided with a file containing three independent documents and clear content separations. GPT-5c features the lowest relative and total error rate. However, hallucinations occur more often than in GPT-5e. If this is influenced by the effect of “lost in the middle,” it should be subject to further investigation. However, we believe the “lost in the middle” issue may be attributed to the poor performance of GPT-5s, as the OPS-catalogue, containing a straightforward list of all encodable codes on 662 pages, does not provide an attention mechanism focusing on an LLM's reasoning. 28 Another variant to improve the code extraction abilities of GPT is an approach according to which codes are not generated, but only confirmed. 29

When utilizing an LLM for complex tasks, data hallucinations frequently occur. 30 Using its transformer architecture, a complex multi-layered network of relationships between words and phrases is generated on which the most probable next word is determined. 31 If insufficient context is available as a foundation for reasoning, the determined most probable word may not correspond to the context or may even be fictional. 32 The main pitfall in practical application is failing to recognize hallucinations as such without verification. In our data, misuse and nonsense hallucinations occurred with comparable frequency. While nonsense hallucinations are easy to detect due to the missing code equivalent, misuse hallucinations are prone to cause miscoding. First attempts at detecting hallucinations are under research using semantic entropy. 33 Our data strongly support the thesis that contextualization significantly reduces hallucinations and thus leads to a great increase in quality. We observed indications for a greater influence of detailed, universally applicable rules on that specific topic as opposed to the provision of example sentences, following the principle of RAG-enhancement. 34 Besides that, there are promising strategies for hallucination mitigation, like “chain-of-verification” prompting strategies, post hoc code rule-based justification and consistency checks. 35 Future studies should focus on the analysis of different hallucination mitigation strategies.

A limitation of this study is the testing of OPS-code extraction based on a single LLM focusing on a single procedure. Future studies should aim for an intermodal platform comparison based on the findings of this study. Regarding the definition of correct or incorrect coding, discrepancies were resolved by consensus with our professional coders on an individual basis, not following a predefined protocol. We acknowledge that the usage of closed, continuously updated models may cause variability over time despite identical input and prompting.

Due to the exploratory character of the study, no formal correction for multiple testing was chosen. Therefore, marginal *p* -values are to be interpreted with caution. After previously demonstrating the general feasibility of LLMs in OPS-code extraction from medical reports, 19 we were able to demonstrate the influence of different CE-modalities on LLMs' code extraction abilities.

A potential direction for future development lies in the use of domain-specific models, which may achieve high precision in well-defined application areas. 36

However, before clinical application, great research is required focusing on generalizability and local applicability. In the future, independent, locally operating interactive LLM-based agents may be developed to be integrated into the hospital's digital infrastructure.

## Conclusion

Our single-center proof-of-concept study demonstrates a significant improvement in GPT-5 OPS-code extraction ability from ORs in meningioma surgery by implementing generalizable topic-specific CE.

## Notes

**Clinical Relevance Statement**
Accurate OPS coding is crucial for correct DRG assignment and thus for the economic sustainability of surgical departments. The use of LLMs to support OPS-coding may standardize coding quality, reduce the time burden on surgeons, and minimize coding errors. This has the potential to streamline clinical workflows, improve resource utilization, and increase revenue security within the DRG-based reimbursement system. In the context of increasing economic pressure, AI-assisted coding may represent a sustainable strategy to optimize surgical care delivery.

## Notes

**Multiple-Choice Questions**

1. What was the primary objective of this study?
  - To compare GPT-5 with conventional hospital coding software for DRG encryption.
  - To evaluate the feasibility of OPS-coding by human coders versus AI coders.
  - To assess how different modalities of context enhancement affect GPT-5s coding accuracy.
  **Correct Answer** : The correct answer is option c. The study evaluates the effect of different modalities of context enhancement to GPT-5s OPS-code extraction ability from operatie reports of meningioma surgery.
2. Which context-enhancement strategy resulted in the highest OPS-coding accuracy?
  - GPT-5o
  - GPT-5e
  - GPT-5c
  - GPT-5r
  **Correct Answer** : The correct answer is option c. The combination of the OPS-Catalogue, Coding Rules and Exemplary Phrases led to the highest rate of correct coding.
3. What was the definition of hallucination in this study?
  - As incorrect use of existing codes or usage of nonexisting codes.
  - As any incorrect OPS-code.
  - A vital mistake leading to MDK rejection.
  **Correct Answer** : The correct answer is option a. This study defines misuse hallucinations, in which existing codes are incorrectly combined with ficitve content, as well as nonsense-hallucinations, in which a fictive code is applied.

## Declaration of GenAI Use

The authors declare the use of generative AI in the research and writing process. According to the GAIDeT taxonomy (2025), the following tasks were delegated to GAI tools under full human supervision: the authors used ChatGPT to extract OPS-coding from operative reports that were analyzed in this work. No AI assistance was used for writing this manuscript. The GAI tool used was GPT-5. Responsibility for the final manuscript lies entirely with the authors. GAI tools are not listed as authors and do not bear responsibility for the final outcomes.
