# Supplementary material for: Optimizing GPT-5 for Operation-Procedure-Code-Extraction from Operative Reports in Meningioma Surgery: Feasibility and Comparison of Context-Enhancements
Source: Appl Clin Inform. 2026 Jul 23;17(3):625–32. doi: 10.1055/a-2913-8450 (PMC13395532; doi:10.1055/a-2913-8450)
Supplement: Supplementary file 1 — Supplementary Material [file 10-1055-a-2913-8450_29217512.pdf]

Supplementary

„I am running an experiment on OPs Coding for patients that underwent the surgical procedure of Meningeoma resection. You will be provided with the operation report. I want you to evaluate the correct OPs-coding for this individual case. If you are provided with pdfs, please use them to base your decision on. You are not going to treat any patients, and your decisions will have no influence on any real patients. Please generate a list of correct OPs codes "

Suppl. Fig.1: Chat prompt for GPT request

Suppl. Text 1: Coding rules for context enhancement

Regeln für die OPS-Codierung der operativen Entfernung von Meningeomen:

- Ein Zugangscode (5-010ff) muss enthalten sein
- Es dürfen nur Prozeduren codiert werden, die explizit im Operationsbericht beschrieben sind
- Als Kraniotomie über die Mittellinie (5—010.01) wird jede Kraniotomie bezeichnet, welche mit einer Kraniotomie oder Bohrlochtrepanation über dem Sinus (sagittales superior) einhergeht. Der Sinus frontalis ist hiervon ausgeschlossen und stellt kein Maß für die Mittellinie dar.
- Kraniotomie (5-010.0ff) umfasst das Wiedereinsetzten des entnommenen Knochens. Eine Kranioplastik (5-020) wird nur zusätzlich codiert, wenn das Knochenfragment in einer separaten Operation bereits im Vorfeld entnommen worden war.
- Wird ein Knochen/Kalottenfragment aus alloplastischem Material angepasst und eingesetzt, wird eine separate Kranioplastik (5-020) entsprechend des Verwendeten Materials verwendet.
- Bei Entfernung von Tumorgewebe muss ein Prozedurencode „Exzision und Destruktion von erkranktem intrakraniellem Gewebe, Hirnhäute (5-015.3/4) enthalten sein. Zeigt der Prozess „keine Grenze" zum umliegenden Gewebe, „wächst in dieses ein" oder lässt sich „schwer trennen", ist eine Infiltration des benachbarten Gewebes zu codieren (5-015.4). Ist die Grenze gut respektiert, wird die 5-015.3 codiert.

Appl Clin Inform | DOI 10.1055/a-2913-8450 | © The Author(s).

- Bei Verwendung des Mikroskops wird „mikrochirurgische Technik“ (5-984) codiert
- Bei Verwendung einer Neuronavigation wird Anwendung eines Navigationssystems, radiologisch“ (5-988.0) codiert.
- Bei Verwendung von Ultraschall wird Anwendung eines Navigationssystems, sonographisch“ (5-988.2) codiert.
- Bei Verwendung von intraoperativem Neuromonitoring, SEP, MEP wird „Intraoperatives neurophysiologisches Monitoring“ (8-925.01/21/31) je nach angegebener Dauer verwendet.
- Bei Verwendung direkter Stimulation auf dem Cortex wird die 8-925.00/20/30 je nach angegebener Dauer verwendet.
- Gibt es keine explizite Angabe zur Verwendung oder der Dauer des Neuromonitorings, muss die Verwendung und die Dauer des Neuromonitorings explizit durch das LLM erfragt werden.
- Bei Aufkleben oder Abdichten der Dura durch Muskel, Fett, Faszie, Periost oder Tachoseal, Lyoplant, Hämopatch o.ä. wird „Rekonstruktion der Hirnhäute“ (5-021.ff) codiert.
- Wird eine Dura ausschließlich durch eine wasserdichte Primärnaht genäht und nicht geklebt, bzw. keine zusätzlichen Materialien zur Abdichtung verwendet, wird keine „Rekonstruktion der Hirnhäute“ (5-021.ff) codiert. Rekonstruktionen der Hirnhäute im Rahmen eines Wundverschlusses nach transkranieller Operation werden mit 5-021.0 codiert. 5-021.1/2 sind Eingriffen mit Durarekonstruktion nach Trauma, Entzündung, Eröffnung des Sinus frontalis oder Z.n. transnasalem Eingriff vorbehalten.
- Bei Vorliegen eines Rezidivs wird „Re-Operation“ (5-983) codiert
- Die Anlage von Drainagen wird nicht codiert
- Die Verwendung von blutstillenden Maßnahmen oder Wirkstoffen wird nicht mitcodiert

<u>English translation of rules for OPS coding of surgical removal of meningiomas:</u>

- An approach code (5-010ff) must be included
- Only procedures that are explicitly described in the surgical report may be coded

- A midline craniotomy (5—010.01) is defined as any craniotomy that involves a craniotomy or burr hole trepanation involving the sinus (sagittal superior). The frontal sinus is excluded from this and does not represent a measure of the midline.

- Craniotomy (5-010.0ff) includes the replacement of the removed bone. Cranioplasty (5-020) is only coded additionally if the bone fragment had already been removed in a separate operation beforehand.

- If a bone/calotte fragment made of alloplastic material is fitted and inserted, a separate cranioplasty (5-020) is used according to the material used.

- When removing tumor tissue, the procedure code “Excision and destruction of diseased intracranial tissue, meninges (5-015.3/4)” must be included. If the process shows “no margin” to the surrounding tissue, “grows into it” or is “difficult to separate”, infiltration of the adjacent tissue must be coded (5-015.4). If the margin is well respected, 5-015.3 is coded.

- If a microscope is used, “microsurgical technique” (5-984) is coded.

- If neuronavigation is used, “use of a navigation system, radiological” (5-988.0) is coded.

- If ultrasound is used, “use of a navigation system, sonographic” (5-988.2) is coded.

- When using intraoperative neuromonitoring, SEP, MEP, “Intraoperative neurophysiological monitoring” (8-925.01/21/31) is used depending on the specified duration.

- If there is no explicit information on the use or duration of neuromonitoring, the LLM must explicitly inquire about the use and duration of neuromonitoring.

- If the dura is sealed using muscle, fat, fascia, periosteum, or Tachoseal, Lyoplant, Hemopatch, or similar, “reconstruction of the meninges” (5-021.ff) is coded.

- If the dura is sutured exclusively with a watertight primary suture and not glued, or if no additional materials are used for sealing, “reconstruction of the meninges” (5-021.ff) is not coded. Reconstruction of the meninges as part of wound closure after transcranial surgery is coded as 5-021.0. 5-021.1/2 are reserved for procedures involving dura reconstruction after trauma, inflammation, opening of the frontal sinus, or following transnasal surgery.

- In the event of a recurrence, “reoperation” (5-983) is coded.
- The insertion of drains is not coded.
- The use of hemostatic measures or agents is not coded.

Suppl. Text 2: Phrases triggering code for context enhancement

OPS-Code	Phrase
5-010.00	-Setzen von Bohrlöchern, Ausfräsen eines ovalären Knochendeckels.<br>Es erfolgt eine ca. 1,5 cm messende Kraniotomie über eine Distanz von 4 cm, ab ehemaliger Trepanationskante.<br>-Hautdesinfektion und sterile Abdeckung sowie Setzen eines geraden paramedianen Hautschnittes rechts hemisphäriell und Zurückschlagen von Galea-Periost. Darstellung der Kalotte und kreisrunde Kraniotomie parietal rechts.<br>-3 Bohrlochtrepanationen, Aussägen eines von frontal nach temporal reichenden, annähernd ovalären Knochendeckels.<br>-Nach Lösen der Fixationsschrauben kann der Knochendeckel als auch das Mesh nach Rekraniotomie problemlos luxiert werden.
	-Bohrlochtrepanation und Kraniotomie von ca. 6 x 6 cm<br>-Dementsprechende Anlage von Bohrlöchern und Herausnahme eines links parietalen Knochendeckels<br>-Einbringen eines Bohrloches auf dem dorsalen-medialen Rand der geplanten Kraniotomie. Durchführen der ca. 5 x 6 cm großen Kraniotomie.<br>-Herausfräsen des Knochendeckels im Sinne einer erweiterten frontolateralen Kraniotomie.<br>-Ansetzen von drei Bohrlöchern und anschließende Kraniotomie, Elevation des Knochendeckels.<br>-Bohrlochtrepanation temporal und parietal. Ausfräsen eines ca. 7 x 7 cm messenden Knochendeckels.<br>-Planung eines ca. 10 cm langen, geraden Schnittes frontal rechts. -Bohrlochtrepanation am okzipitalen Rand und Ausfräsen eines ca. 7x7 cm messenden Knochendeckels.
	-Bohrlochtrepanation und Kraniotomie.<br>-Zwei Bohrlochtrepanationen, Ausfräsen eines annähernd ovalären Knochendeckels.
	-Ansetzen von drei Bohrlöchern und anschließende Kraniotomie,<br>-Darstellung der Kalotte und nachfolgende rechtsfrontale Kraniotomie.<br>-Anlage eines Bohrloches und Herausnahme eines rechts parietalen Knochendeckels<br>-nachfolgende rechtsfrontale Kraniotomie unter Kontinuitätserhalt der Stirnhöhle bzw. ohne Überschreiten des Sinus sagittalis superior.<br>-Nachfolgend in etwa 1 cm Abstand Umrunden des tastbaren Befundes mit dem Kraniotom, nachdem mit dem Bohrloch im Keyhole frontotemporal ein Zugang gesetzt worden ist.<br>-Nachfolgend Setzen von 3 Bohrlöchern. Ausfräsen eines ovalären Knochendeckels, welcher abgegeben und für die spätere Restitution mit Mikroplatten und Mikroschrauben versehen wird.
	-Darstellung der Schädelkalotte und kreisrunde Kraniotomie<br>Fissur. Das Bohrloch wird in loco typico im frontotemporalen Übergang als Keyhole gesetzt. Nachfolgend Entfernen des Knochendeckels.<br>-Bohrlochtrepanationen. Ausfräsen eines annähernd ovalären Knochendeckels.<br>-Anlage von zwei Bohrlöchern, der eine wird am Übergang frontotemporal eingesetzt, der zweite lateral davon temporal.<br>-Bohrlochtrepanation am orbitozygomatischen Punkt sowie weiter dorsal vor der Schnittführung. Herausfräsen des Knochendeckels unter Exploration des medialen Keilbeinflügels sowie der frontale als auch Temporalregion.

	-Freilegung der links frontalen Kraniotomie
5-010.01	-Bohrlochtrepanation temporal und auf den Sinus sagittalis superior.<br>-Setzen zweier Bohrlöcher an den Sinus sagittalis superior. Erstellen einer ovalen Kraniotomie.<br>-Bohrlochtrepanation apikal der Linea temporalis rechts sowie auf dem Sinus sagittalis superior. Fortlaufende Kraniotomie bis vor den Sinus frontalis frontobasal paramedian rechts<br>-Nun erneute Identifikation der Sagittalnaht und des darunterliegenden Sinus sagittalis superior. Auch mit der Navigation Setzen von zwei Bohrlöchern an der ventralen und dorsalen Grenze der Tumorausdehnung. Durchführen einer Kraniektomie im Durchmesser von knapp 5 cm.<br>-Falxmeningeom. Bohrlochtrepanation am mediookzipitalen Rand der geplanten Kraniotomie auf der Sutura sagittalis.<br>-Es erfolgt nun die Bohrlochtrepanation auf dem Sinus sagittalis superior sowie weiter lateral. Nach Darstellen der Dura Herausfräsen eines ca. 8 x 8 cm messenden Knochendeckels.<br>-Es erfolgt auf den Sinus und bds. lateral direkt unter den Muskel, der dafür knapp abgeschoben wird, die Bohrlochtrepanation unter reichlicher Spülung. Die letzte Knochenlamelle wird mit dem Dissektor entfernt, dann erfolgt die Kraniotomie bds..<br>-linken hochfrontalen Parasagittalregion. Anlage von Bohrlöchern und Herausnahme eines links parasagittal bis an den Sinus heranreichenden Knochendeckels.<br>-Nachfolgend Einschneiden eines bogenförmigen Hautschnittes frontotemporal, im Sinne eines erweiterten pterionalen Zugangs mit Übergang über die Mitte.<br>-Die Kalotte ist exploriert, es erfolgt die Bohrlochtrepanation auf dem Sinus sagittalis superior. Darstellen der Dura und Herausfräsen eines ca. 6 x 6 cm messenden Knochendeckels.<br>-Falxmeningeom. Navigationsgestützte Kraniotomie über 2 mediane und 1 laterales Bohrloch.<br>-Nun 2 Bohrlochtrepanationen auf den Sinus sagittalis superior. Ausfräsen eines Knochendeckels über die Mittellinie nach links ca. 1 cm reichend, nach rechts ca. 3 cm reichend.<br>-Falxmeningeom, Dann erfolgt die erneute Kraniotomie des großen Deckels<br>parasagittal mit Beziehung zum Sinus sagittales superior. –<br>-Dementsprechende Anlage von Bohrlöchern und Herausnahme eines bis an den Sinus sagittalis heranreichenden rechts parasagittalen Knochendeckels.
5-010.03	-Herausnahme eines rechts temporalen Knochendeckels<br>-Anlage von Bohrlöchern und Herausnahme eines links temporalen Knochendeckels, der nach Säuberung zur Restitution in feuchten Kompressen aufbewahrt wird.<br>-Anlage von Bohrlöchern und Herausnahme eines rechts subokzipitalen Knochendeckels
5-010.04	-Anlage von Bohrlöchern und Herausnahme eines rechts subokzipitalen Knochendeckels<br>-Am vorderen unteren Rand und nach Navigationsortung auf dem Felsenbein links am okzipitalen Rand werden Bohrlochtrepanationen gesetzt. Dann Ausfräsen eines annähernd dreieckigen Knochendeckels mit einer breiten Basis zum Ohr hin.<br>-Anlage von Bohrlöchern und Herausnahme eines rechts subokzipitalen Knochendeckels<br>-Nachfolgend Anlage einer Bohrlochtrepanation im Sinuswinkel zwischen Sinus transversus und Sinus sigmoideus. Von dort ausgehend entsprechende Kraniotomie über 3 x 3 cm.<br>-subokzipitalen Zugang. Der Sinus transversus und der Sinus sigmoideus kommen eindeutig zur Darstellung.<br>-Bohrlochtrepanation leichtgradig unter dem Sinus Transversus sowie weiter basal. Herausfräsen eines ca. 5 x 5 cm messenden Knochendeckels,
5-011.0	-Hier erfolgt die Orbitotomie mit der Piezo-Säge, das Fragment wird asserviert.
5-015.3	-Das Meningeom lässt sich gut vom gesunden Kortex abgrenzen.

	-Dieser hat nur an 2 Stellen arachnoidale Verklebungen, welche nach Koagulation durchtrennt werden können. Letztendlich ist der Tumor samt Duraansatz komplett reseziert.<br>-Das Meningeom wird nach kaudal verfolgt und schlussendlich nach vollständiger Durchtrennung aller zuführenden Gefäße und Präparation vom Cortex aus dem Situs genommen.<br>-Ausdehnung des duraanliegenden, extraaxialen Tumors. Durch die intratumorale Verkleinerung mit dem CUSA und anschließender sukzessive Kapselpräparation kann der Tumor mikroskopisch in toto entfernt werden (Simpson Grad I).<br>--rechts frontobasale Raumforderung, verdächtig a.e. auf ein Meningeom.<br>-Es zeigt sich eine zunächst gute arachnoidale Grenzschicht, welche vorsichtig präpariert wird.<br>-der Verdacht eines ausgedehnten Meningeoms rechts. Nun Schrittweise Darstellung der Tumor/Arachnoideaschicht und Freipräparation des Tumors in alle Richtungen.<br>-V.a. auf ein präzentral gelegenes, raumforderndes Konvexitätsmeningeom. Mit Sauger und Bipolar kann eine deutliche piale Trennschicht zum intakten Kortex dargestellt werden, dieser ist nicht infiltriert.<br>-seit einigen Monaten ein inzidentelles Falxmeningeom frontal linksseitig bekannt. Anschließend wird die Tumorresektion mit dem Lösen des Meningeoms entlang der intakten Arachnoideagrenze begonnen.<br>ein Meningeom links frontal. Freilegen des Tumors und vorsichtige Präparation vom Kortex.
	-Vorstellung des Patienten erfolgte wegen eines Frontalhirnmeningeom<br>-Die kernspintomografischen Charakteristika legen ein Meningeom, am ehesten vom Felsenbein ausgehend, nahe. Die Tumorkapsel lässt sich in der arachnoidalen Verschiebeschicht schrittweise präparieren<br>-Es finden sich Meningeomanteile. Am Tentorium hängt der Tumor, dieser kann aus dem Arachnoideabett geborgen werden.<br>-Die kernspintomografischen Charakteristika legen ein Meningeom, am ehesten vom Felsenbein ausgehend, nahe. Die Tumorkapsel lässt sich in der arachnoidalen Verschiebeschicht schrittweise präparieren<br>Eine daraufhin initiierte MR-Diagnostik des Schädels verweist auf ein Planum sphenoidale-Meningiom. Das Meningeom selbst haftet breitflächig im Bereich der Frontobasis an und lässt sich mikrochirurgisch und z.T. makroskopisch sowohl gut identifizieren wie auch nachfolgend entfernen. Eine Infiltration im Bereich der Sehnerven lässt sich nicht verifizieren.<br>-Seit dem ersten Quartal war bei der Patientin eine Raumforderung im Felsenbein/Tentorium der rechten Seite bekannt, am ehesten einem Meningeom entsprechend. Kaudal lässt sich die Tumorgrenze sehr schön, auch um den Tumor herum, nachweisen.<br>-Als Ursache war das ca. 17 x 16 mm große Meningeom des Planum sphenoidale in einem MRT dargestellt. Einsenden eines Schnellschnittpräparates, dieses ergibt die Diagnose eines Meningeoms. die Kapsel des Tumors ist erhalten<br>-allerdings zeigt sich der V.a. eine Meningeomerkrankung. Hierbei gelingt es, sämtliche Tumoranteile im Bereich der Temporalregion zu resezieren und den Tumoransatz sowohl mittels Fräse als auch Bipolator zu veröden und ebenso zu beseitigen.<br>-darüber hinaus ein Größenwachstum des meningeomverdächtigen Gewebes auf ca. 3 x 3 cm,<br>-In der Bildgebung hatte sich ein ausgedehntes Meningeom um die rechte MCA-Gruppe herum in einer Größe von 6,5 cm gezeigt. Schritt für Schritt kann der Tumor aus dem Gewebebett unter Beachtung der arachnoidalen Schicht herausgehoben und reseziert werden.<br>-Es präsentierte sich hier ein ausgeprägtes Keilbeinflügelmeningeom rechts. eine arachnoidale Grenzschicht ist erhalten.<br>-Bildmorphologisch ist hier sicherlich am ehesten von einem Meningeom auszugehen. Kaudal kommen letztendlich die kaudalen Hirnnerven zum Vorschein, die sich ein jedoch durch eine arachnoidale Schicht vom Tumor genau abgegrenzt ist.<br>-Nach Lösen einiger arachnoidaler Briden und Abschieben der Arachnoidea lässt sich am Keilbeinflügel der typisch bräunlich-rötliche meningeale Tumor darstellen.

5-015.4	-zeigt der Tumor in seinen zentralen Anteilen unmittelbar über den präzentralen Kortex eine erhebliche Anheftung und Infiltration in das darunterliegende Kortexgewebe.<br>-Die Schicht zum Hirngewebe respektiert nicht die Arachnoidea. Die Tumorkapsel jedoch haftet am N. opticus an.<br>-vorsichtige Präparation des Tumors entlang der Frontobasis. Hier zeigen sich jedoch ausgiebige starke Verwachsungen<br>-In dieser Umgebung respektiert der Tumor die arachnoidale Grenzschicht nicht und wird aus dem Kortexgewebe präpariert.<br>-Am medialen und dorsalen Rand wird das Hirngewebe vom Tumor infiltriert.
	-Resektion des sphenoorbitalen Meningeoms<br>-zeigte sich der V.a. ein Meningeom. Der Tumor respektiert in Gänze die Arachnoidea.<br>-Komplikationslose Kraniotomie und Resektion von drei Konvexitätsmeningeome temporo-parietal links. Makroskopisch imponiert vor allem ein, die Meningeome überschreitender Tumorrasen, welcher nahezu der kompletten Dura von innen anhaftet. Simpson Grad 1<br>-Die Bildgebung des Kopfes hatte ein ca. 5 x 6 cm großes Meningeom rechts parietal ergeben. Ablösen des Meningeoms. Dieses hat die Arachnoideagrenze zum Gehirn zumeist nicht respektiert.<br>-Bildmorphologisch ist hier jedoch sicherlich am ehesten von einem Meningeom auszugehen. Letztendlich wird die Dura entlang der Tumorränder eröffnet, der Tumor selbst quillt leichtgradige vor. Die Ränder zeigen zunächst eine arachnoidale Verklebung sodass sie unter Koagulation und infolge scharfer Dissektion mit der Schere die Grenze dargestellt werden muss.<br>-mit Nachweis einer meningeomverdächtigen Raumforderung rechts frontotemporal. Nun werden die Ränder des Tumors zur Arachnoidea freipräpariert sowie die teils V.a. frontal infiltrierte Kortexanteile freipräpariert.<br>-V.a. ein Olfaktoriusrinnenmeningeom frontal. Der rechte N. olfactorius ist in dem Tumor verwachsen und kann nicht erhalten werdenvvvvvvv postzentral gelegene Konvexitätsmeningiom weiter größenprogredient. Ein minimaler bindegeweblicher Rest wird im Bereich der größeren Brückenvene belassen, um keine Verletzung der Venenwand zu provozieren.<br>-legt die Diagnose eines parasagittalen Meningeoms nahe. Die Tumorkapsel infiltriert partiell das ödematös veränderte, perifokale Hirngewebe und es kann nicht durchgehend die Arachnoidea erhalten werden<br>-Diagnose eines Meningeoms. Weiteres Abschieben des teilweise infiltrativ wachsenden, extraaxialen Tumors, der die Arachnoidea nicht allseits respektiert.<br>-Schnellschnitt: Meningeom. Der Tumor wird auch dort in Gänze herausgelöst und unter Hilfsmitteln des CUSAs aber auch der scharfen Abtrennung entfernt.<br>-atypisches Meningeom. Zirkumferentes Präparieren des etwas festeren Gewebes, das rosa fluoresziert, aus dem umgebenden gelblichen, nicht fluoreszierenden Gewebe.<br>-Ursächlich hierfür findet sich ein der Falx anliegender Befund im Bereich der linken Zentralregion welche am ehesten einem Meningeom. Nun vorsichtige arachnoidaler Präparation der Tumorgrenze was im ventralen Bereich nicht gelingt, hier muss zunächst koaguliert werden um eine Präparationsschicht zu halten.<br>-Falxmeningeom. Hier zeigt er sich bereits nach wenigen Millimetern in der Tiefe infiltrativ ins umliegende Gewebe wachsend
	-V.a. ein ossär infiltriertes Meningeom<br>-V.a. ein ausgedehntes Keilbeinflügelmeningeom. Die Tumorpräparation erfolgt subtil unter Wahrung der Tumorgrenze, bis schlussendlich der Tumor in Gänze reseziert ist.<br>-Nachfolgend Präparation des Chiasmas bis etwa zur Mitte und --Dekompression derselben. Nunmehr im Bereich der Schädelbasis --Entlastung und Entnahme von einzelnen meningealen verdächtigen Tumornestern. Der Rest wird im Sinne einer Simpson-Grad II-Resektion koaguliert.

	-In der nachfolgenden Diagnostik war die Diagnose des Keilbeinflügelmeningeoms rechts gestellt worden. Zum Temporalpol hin ist die Arachnoidea des Gehirns nicht respektiert.<br>-neuerlichen Rezidivs bei gesichertem Meningeom WHO Grad II. Zum Teil ist der Tumor infiltrativ in das Nachbargewebe eingewachsen<br>-Es bestehen starke bridige Verbindungen und eine piale Gefäßversorgung aus dem frontalen Cortex heraus.<br>-Falxmeningeom, Infiltration der Galea<br>bekannten rechtsseitigen frontalen Meningeoms. . Die Arachnoidea ist überwiegend erhalten. An wenigen Stellen konnte sie aufgrund des invasiven Wachstums des Meningeoms nicht erhalten bleiben.
5-016.2	-Entfernung des infiltrierten Knochens.<br>… unter Miterfassung des infiltrierten Knochens.
5-020.61	-Es wird nun der Trepanationsdefekt mit einer entsprechend modellierten Palacos-Plastik gedeckt.<br>-Rekonstruktion der Orbita (Orbitadach sowie laterale Orbitawand) mit einem 3-D-Mesh, welches nach Anmodellieren mit Minischrauben fixiert wird,<br>-Diese dichtet sehr gut ab. Nachfolgend wird nunmehr der Palacosdeckel angepasst sowie mit drei Platten fixiert.<br>-Der Bereich der Kalotte, der ausgesägt und mit Tumor infiltriert worden ist, wurde dann mit Palacos ersetzt.<br>-Der osteoklastische knöcherne Defekt im Bereich des Keilbeinflügels nach temporal wird mit einem Titanmesh überdeckt, welches ebenfalls mit Minischrauben fixiert wird.
5-021.0	-Bei Persistenz des Liquorflusses Einsatz von Tachosil im Sinne einer Duraplastik.<br>-Lyoplant wird aufgetragen. Anschließende Aufbringung von Tachosil. Dieses ist jedoch nicht ausreichend, groß sodass die restlichen Stellen mit einem Haemo- Patch abgedichtet werden.<br>-Aufbringen von Tachosil auf die Duranaht.<br>-Bei sicherer Bluttrockenheit erfolgt die Duranaht, residuelle Duradehiszenz werden mit Lyoplant-Onlay verschlossen<br>-Auflage von Tachosil.<br>-Wasserdichter Duraverschluss mit Duraplastik mit vorbereitetem Galea-Patch und Anlage von zwei Tachosil-Streifen an die Sutur.<br>-Hier erfolgt eine Duraplastik mit Tachosil.<br>-Nach Einpassung des Lyoplant-Onlays wird dieses darüber hinaus mit Tachosil abgedichtet.<br>-Es erfolgt eine Duraplastik mittels Lyoplant-Onlay.<br>-Bei Bluttrockenheit erfolgt das Ankleben eines Lyoplant-Onlay mit Kontakt zur Dura in allen Richtungen und Fixierung der Lage mittels Tachosil<br>-Ausgedehntes Anfertigen einer Duraplastik mittels Galea und Alloplast.<br>-Bei weiterhin bestehendem, großen Duradefekt wird die Duraplastik um Auflage von Lyoplant Onlay ergänzt.<br>-Bei weitgehend aufgebrauchter Dura und deutlich bestehenden Duradehiszenz, erfolgt eine Duraplastik mit Lyoplant-Onlay.<br>-Der Duradefekt wird mittels Lyoplant-Onlay gedeckt.<br>-Es verbleibt zirkumferent eine Lücke von 2 bis 5 mm. Hier wird Tabotamp aufgelagert, dann Tachosil zur Sicherung aufgelegt<br>-Dies wird an einigen Dura Rändern mittels Einzelknopfnaht fixiert, ist jedoch nicht ausreichend und eine komplette Abdichtung zu erreichen. Somit wird ein Lyoplant-Onlay aufgebracht.<br>-Bei Bluttrockenheit erfolgt das Ankleben eines Lyoplant-Onlay mit Kontakt zur Dura in allen Richtungen und Fixierung der Lage mittels zwei Einzelknopfnähten aber auch bei kleineren Defekten mittels Tachosil<br>-Dann wird die Dura wieder verschlossen, dies gelingt nicht vollständig, sodass zum wasserdichten Verschluss ein Tachosil aufgelegt werden muss.<br>-Duraverschluss mittels fortlaufender Duranaht unter Zuhilfenahme von Galea-Periost und bei Persistenz des Liquorflusses mittels Tachosil (Duraplastik).<br>-Bei Bluttrockenheit Einbringen einer Lyoplant-Duraplastik.

	-Bei Bluttrockenheit erfolgt abschließend der Duraverschluss durch Einbringen einer Dura-Onlay-Plastik, die liquordicht eingebracht wird.<br>-Liquordichtes Überkleben mit Tachosil.<br>-Die Dura wird mit multiplen Haltefäden an die intrakraniellen Reste vernäht, sodass eine Auflage für ein Lyoplant-Onlay 7,5 x 7,5 cm groß entsteht.<br>-Bei allgemeiner Bluttrockenheit Auffüllen der Resektionshöhle mittels physiologischer Kochsalzlösung und neuerlicher Duraverschluss unter Zuhilfenahme von Galea-Periost bzw. bei Persistenz des Liquorflusses mittels Tachosil (Duraplastik).<br>-Es gelingt aber nicht vollständig, sodass man noch Duraersatzmaterial (Lyoplant-Onlay) auflegt sowie etwas Tachosil an dem Randbereich.<br>-Mit 5.0er-Fäden wird die Neodura ausgenäht, beim Versuch einer fortlaufenden Naht reißt sie ein. Es wird daraufhin entschieden, wiederum Lyoplant-Onlay aufzubringen.<br>-Der Duradefekt kann mit dem zuvor präparierten Periost nicht ausreichend gedeckt werden, sodass Lyoplant-Onlay verwendet wird. Nunmehr Auflage von Tissue-Dura im Bereich der resezierten Dura unter Beachtung der Grenzen.<br>-Die Dura wird nunmehr wieder rekonstruiert und mit Tachosil ergänzt, um so einen wasserdichten Verschluss bei fehlenden Alternativen zu gewährleisten.<br>-Schließlich basal und kranial Auflagern von Lyoplant-Onlay<br>-Fortlaufende Duranaht und Aufbringen eines großen, in Streifen geschnittenen Tachosils.<br>-Neuerlicher Duraverschluss unter Verwendung von Galea-Periost bzw. bei persistierendem Liquorfluss unter Prävention einer Liquorfistel mittels Tachosil (Duraplastik).<br>-Zurückankern der Dura, fortlaufende Duranaht. Auflegen von Tachosilplatten auf die Duranaht, im Bereich des Sinus sagittalis war sonst keine ausreichende Blutstillung zu erreichen.<br>-bei persistierender Liquorfistel mittels Tachosil im Sinne einer Duraplastik.<br>Aufgrund der noch bestehenden Defekte wird ein Periostlappen eingeschwenkt und nachfolgend Duraersatzmaterial mit Tachosil aufgetragen, da dieser nicht komplett wasserdicht abdeckt.<br>-Die entstandenen Dehiszenzen werden mit dem präparierten Periost als auch Tachosil abgestellt.<br>-Duraverschluss mit fortlaufender Duranaht. Liquordichtes Verkleben mit Tachosil.<br>erfolgt der liquordichte Duraverschluss mit einem Lyoplant-Onlay.<br>-Dieses wird nun mit Tachosil überklebt. Bei teils resezierter Dura erfolgt die Duraplastik mittels Lyoplant-Onlay.
5-021.2	-Das aufgedehnte Foramen ethmoidale zeigt an seinem Boden einen hellen Schimmer, welcher am ehesten einer feinen Membran entsprechen könnte. Die Dura ist eindeutig gefenstert.<br>-Dann zunächst Einschieben von betaisodonagetränkten Gelaspon zum Verschluss des Sinus frontalis. Nachfolgend Auflage einer Schicht Tachosil in diesem Bereich.
5-021.3	-Einbringen einer Lyoplant-Onlay-Duraplastik. Subokzipitalregion. petroclivalen Meningeom.<br>-subokzipitalen Zugang. Es erfolgt der Duraverschluss mit einem Lyoplant-Onlay, welches liquordicht mit Tachosil überklebt wird.<br>-Es wird ein Lyoplant-Onlay bei dem kompletten Duraverschluss aufgelegt nach retrosigmoidalem Zugang.
5-983	-Wiedereröffnen der Schnittführung,<br>-größenprogredienten Rezidivtumor<br>-Rasur der Kopfhaut im Bereich des ehemaligen koronaren Hautschnitts links frontal. Die Dura war bei der Erstoperation mit dem Meningeom reseziert worden, dass Lyoplant-Onlay-Vlies findet sich in situ und ist vernarbt.<br>-Bei der Patientin waren im Vorjahr mehrere meningeale Anteile gesichert wurden. In der Bildgebung zeigt sich neben dem Befund parietookzipital im Wesentlichen der meningiale Anteil am S. Cavernosus, am medialen Keilbeinflügel mit Kompression des rechten N. opticus.

	-Rezidivs bei gesichertem Meningeom WHO Grad II zur Resektion. Wiedereröffnen des links frontal.<br>-Das neu angefertigte MRT zeigte insbesondere im Bereich des Kleinhirnbrückenwinkels eine tendenzielle Vergrößerung des Tumorvolumens des bekannten Resttumors.<br>-Beide erfüllen die Charakteristika eines Meningeomrezidivs. , die partiell verknöchert ist und Herausnahme des alten Knochendeckels.<br>-Wir stellten die Indikation zur nochmaligen Resektion
5-984	-Mikroskopische Eröffnung der Dura mater.<br>-Einschwenken des Operationsmikroskops<br>-Ausschwenken des Operationsmikroskops.<br>-Einschwenken des Mikroskops<br>-Einschwenken des OP-Mikroskops.<br>-unter mikroskopischer Sicht<br>-Es wird dann das Mikroskop eingeschwenkt<br>-Mikroskopisch ist der Tumor<br>-Unter Sicht des Mikroskops<br>-mikrochirurgisch<br>-dann unter Operationsmikroskopsicht
5-988.0	-Einlesen der Neuronavigation. Mit der Neuronavigation Festlegung der Kraniotomiegrenzen.<br>-Referenzierung der Neuronavigation<br>-Kontrolle des Zuganges mittels Navigation.<br>-…die Neuronavigation referenziert.<br>..-unter Nutzung der Neuronavigation. Referenzierung der Neuronavigation .<br>-Durchführung der Referenzierung der Navigation.<br>-Verifizieren der Navigation. Verifizieren der Tumorgrenzen mittels -Navigation.<br>-Nun wird das Navigationssystem mit Hilfe des Navigationssystemsternes angebracht und die Navigation eingelesen.<br>-Referenzierung der Neuronavigation und navigiertes Planen eines gebogenen Schnittes<br>-Kalibrierung der Neuronavigation<br>-Mit dem Navigationspointer wird an der Galea die Tumorausdehnung in Koronarnahthöhe links parasagittal abgefahren<br>-Durchführung der Referenzierung der Navigation. Mit dem Navigationspointer wird der subtemporale Zugang zur Temporobasis angezeigt<br>-Referenzierung der MRT-Daten mit guter Genauigkeit<br>-navigationsgestützte Planung des Zuganges<br>-Indikation zur navigationsgestützten Entfernung. Durchführung der Referenzierung der Navigation<br>-unter Nutzung der Neuronavigation
5-988.2	Mittels Ultraschall Detektion des Tumors und Bestätigung der korrekten Trepanation.<br>Verifizierung der Tumorlage mit intraoperativem USG<br>Mittels Ultraschall Darstellung des Situs<br>Ultraschallkontrolle:<br>Durchführung des transduralen Ultraschalls<br>Durchführen des transduralen Ultraschalls, der über die Kraniotomie erreichbar den echogenen Rezidivtumor zeigt.<br>Einsatz des Ultraschalls. Durchführen des transduralen Ultraschalls.<br>Es erfolgt dann im Ultraschall das Aufsuchen der verschiedenen Tumoranteile
5-988.3	Unter Fluoreszenzlicht zeigt sich eine schwache Fluoreszenz.
8-925.01	unter neurophysiologischem Monitoring gestellt. Anlegen des neurophysiologischen Monitorings.

8-925.01	Anbringen der Elektroden für das intraoperative Monitoring
8-925.01/02	sowie Anbringen der Elektroden für das intraoperative neurophysiologische Monitoring (SSEP, VEP, MEP
8-925.01/02	Anlegen des neurophysiologischen Monitorings (SEP, AEP, N. facialis, N. trigeminus) . 8. Hirnnerv und der 7. Hirnnerv gut stimulierbar
8-925.01/02	Anlegen des neurophysiologischen Monitorings (SEP, AEP, N. facialis, N. trigeminus)
8-925.01/02	Da sich die visuell evozierten Potenziale nach der Präparation etwas verschlechtert hatten
8-925.01/21	Anlegen des neurophysiologischen Monitorings. Während der Operation ist es zu keiner Verschlechterung des SEPs gekommen.
8-925.20	Elektrophysiologisch wird das SSEP abgeleitet.
8-925.21	Anbringen der Elektroden für das IOM (SSEP; MEP; DNS Facialis, Trigeminus).
